# Post-void bladder ultrasound in suspected cauda equina syndrome—data from medicolegal cases and relevance to magnetic resonance imaging scanning

**DOI:** 10.1007/s00264-022-05341-0

**Published:** 2022-02-19

**Authors:** Nicholas Todd, Katerina Dangas, Chris Lavy

**Affiliations:** 1 Newcastle Nuffield Hospital, Newcastle upon Tyne, UK; 2 Nuffield Department of Orthopaedics, Rheumatology, and Musculoskeletal Sciences, University of Oxford, Oxford, OX3 7LD UK

## Abstract

**Objective:**

Post-void residual (PVR) scans of less than 200 ml are increasingly being used to rule out the likelihood of cauda equina syndrome (CES) and to delay emergency MRI scanning in suspected cases. This study was done to review a series of 50 MRI confirmed cases of CES and to test the hypothesis that a PVR of less than 200 ml was unlikely to be present.

**Methods:**

Fifty consecutive medicolegal cases involving CES were audited. Records were reviewed to see if PVR scans were done. MRI scans were reviewed, clinical and radiological diagnosis reviewed, and treatment recorded.

**Results:**

Out of 50 CES cases, 26 had had PVR scans. In 14/26 (54%) the PVR scan was ≤ 200 ml. In one case, the CES diagnosis was in question leaving 13/26 (50%) cases where there was a clear clinical and MRI diagnosis of CES despite the PVR being ≤ 200 ml. All 13 were classified as incomplete cauda equina syndrome (CESI) and all proceeded to emergency decompression.

**Conclusions:**

This study is the first in the literature to demonstrate that there is a significant group of CES patients who require emergency decompression but have PVRs ≤ 200 ml.

The results demonstrate the existence of a significant group of CESI patients whose bladder function may be deteriorating, but they have not yet reached the point where the PVR is over 200 ml. Given the accepted understanding that CESI is best treated with emergency decompression, such patients are likely to have worse outcomes if MRI scanning and therefore surgery is delayed. We recommend the following:

- PVR is recommended as an assessment tool in suspected CES.
- A PVR of ≤ 200 reduces the likelihood of having CES but does not exclude it; clinical suspicion of CES should always lead to an MRI scan.
- Further investigation of PVR as a prognostic tool is recommended.

## Introduction

The CES is a condition that can lead to severe disabling symptoms causing long-term social and medical morbidity. Early diagnosis and treatment of CES can prevent harm. The failure to diagnose and treat CES before there is permanent and/or severe neurological injury is important for all patients and is also important medicolegally. There is no universally agreed definition of CES. Many symptoms and signs are quoted as “red flags” for CES but none reliably predict cauda equina (CE) compression on MR imaging [1–5]. This leads to high rates of negative MRIs in patients who have suspected CES [6]. Bladder ultrasound is a cheap, noninvasive assessment of bladder function, which is widely available in emergency departments. The residual volume of urine present in the bladder post-void (PVR) has been proposed as an accurate assessment of the probability of a patient having CES [6] [7]. Katzouraki et al. have stated that if the PVR is ≤ 200 ml, and there are no clinical signs of CES, the probability of a negative MRI is 98.7%, and such patients do not require emergency MR imaging [7]. Deyo et al. [8] in 1992 went even further and stated that “the predictive value of a negative test (no urinary retention) would be almost 0.9999”.

Whilst we value the assessment of objective measures to diagnose cauda equina syndrome and support the widespread use of bladder scanning, we wish to document our experience that cauda equina syndrome requiring emergency decompression can still be present when a PVR is less than 200 ml.

## Methods

We retrospectively reviewed the two senior authors’ 50 most recent medicolegal reports that concerned patients who were litigating in relation to CES. The reports were prepared for both claimants and defendants. We identified those cases where the PVR was recorded. In cases where the PVR was ≤ 200 ml, we recorded the following: age, sex, bladder symptoms, urinary and/or bowel incontinence at any time, subjective and clinician-tested impairment of perineal sensation, reduced anal tone, level of compression on MRI, confirmation of diagnosis by a radiologist and surgeon, the clinical decision to perform emergency decompression, and the timing of surgery. We used the same criteria as Katzouraki et al. [7] for a positive MRI scan, namely “a large lumbosacral disc prolapse occupying most of the canal cross-sectional area sufficient to compress the CE…*”.*

All cases have been anonymised and have no identifying data in accordance with the World Medical Association Declaration of Helsinki statement of ethical principles for medical research [9].

## Results

**Fig. 1:**
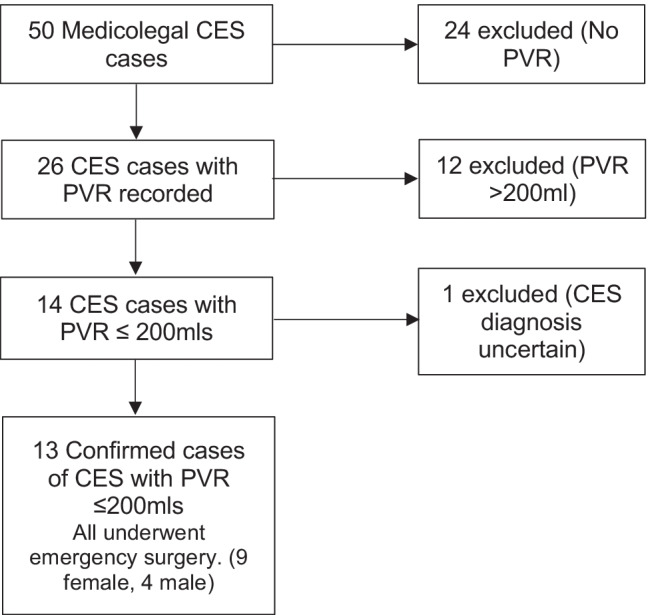
Retrospective analysis of 50 medicolegal cases of CES

These are summarised in the diagram (Fig. 1) and table (Table 1) below. Of 50 medicolegal cauda equina syndrome cases, 26 had records of PVR being measured. In 14 of these, the PVR volume was 200 ml or lower. In one case, there was doubt about the diagnosis, and we therefore excluded it from analysis, leaving 13 cases where there was a clinical diagnosis of CES, confirmed by MRI, and where emergency surgery was performed. In all 13 cases, the cause of cauda equina compression was a lower lumbar disc herniation, eight being at the L4/5 level and five at the L5/S1 level. In all cases, the MRI scan was done within 24 hours of the PVR assessment. All cases were classified as CESI, as there was executive control of bladder emptying, i.e. voluntary voiding was possible and took place before the PVR was measured. In 11 of these cases, surgery was performed within 24 hours of the MRI scan, and in two, there was a delay (2 days and 3 days) despite the diagnosis being clear retrospectively. In many of the cases, there were aspects of treatment that were potentially negligent; however, this aspect of management is not the subject of this review and did not affect the data collected.

**Table 1:** Details of 13 cases of CES with PVR less than 200 ml, who underwent emergency decompression

Case no	Sex	Age	Bladder symptoms	Urine incont	Perineal sensory change		Reduced anal tone	Bowel incont	PVS	MRI confirmed diagnosis	Lesion level	CESI or CESR
					Symptoms	By exam						
1	M	35	Yes	No	Yes	Yes	No	No	14	Yes	L5/S1	CESI
2	F	58	Yes	No	Yes	Yes	Yes	No	30	Yes	L5/S1	CESI
3	F	36	Yes	No	Yes	Yes	No	No	38	Yes	L4/5	CESI
4	F	49	Yes	No	Yes	Yes	No	No	49	Yes	L4/5	CESI
5	M	43	No	No	Yes	Yes	Yes	No	50	Yes	L4/5	CESI
6	F	28	Yes	Yes	Yes	Yes	Yes	No	50	Yes	L5/S1	CESI
7	M	37	Yes	No	Yes	Yes	No	No	74	Yes	L5/S1	CESI
8	F	54	Yes	No	Yes	Yes	Yes	No	135	Yes	L4/5	CESI
9	F	34	Yes	No	Yes	Yes	No	No	140	Yes	L4/5	CESI
10	F	36	Yes	No	Yes	Yes	Yes	No	149	Yes	L4/5	CESI
11	F	39	Yes	No	Yes	No	No	No	166	Yes	L5/S1	CESI
12	F	40	Yes	No	No	No	No	No	169	Yes	L4/5	CESI
13	M	46	Yes	Yes	Yes	Yes	Yes	No	< 200	Yes	L4/5	CESI
Mean		41.1							135			

## Discussion

CES is a constellation of clinical symptoms and signs, not all of which have to be present to make a diagnosis of CES, and many are not present when CES is diagnosed. The syndrome ranges from mild symptoms of CE irritation to severe neurological and visceral injury [10]. Published definitions of CES vary with different symptoms and signs being emphasised by different authors [11]. Early diagnosis and treatment is imperative to achieving good outcomes by preventing further neurological injury and to permit neurological recovery particularly in patients with CESI treated within 48 h [12]. Unfortunately, no symptom or sign or combination accurately predicts CE compression on MR imaging [2–4], which leads to large numbers of negative MRIs performed so that a diagnosis of CES is not missed. A retrospective and then a prospective study of PVR in the assessment of potential CES patients have been performed [6, 7]. The prospective study was of 260 suspected CES patients. A positive MRI was defined as an MRI showing “a large lumbosacral disc prolapse occupying most of the canal cross-sectional area sufficient to compress the CE…*”* The mean canal occlusion was 76.5% (95% confidence interval 72–81%). Emergency MR imaging was performed in 226 patients, 34 (15%) had a positive MRI. Thirty-four patients had normal perineal sensation, normal voluntary anal contraction (VAC) and a PVR ≤ 200 ml. They had deferred MRIs, no MRI was positive, no patient underwent surgery, and none developed CES subsequently. The authors said that in patients with no objective signs of CES and a PVR ≤ 200 ml urgent/emergency, MR imaging is not justified, and MR imaging can safely be deferred to an outpatient basis during normal working hours^7^. In a subsequent letter^12^, the authors said that such patients can be managed on a routine basis “using the local radiculopathy pathway”. Similar symptoms and signs of CES were found in both MRI groups and did not discriminate between the MRI positive and negative patients. In the subsequent letter, it was noted that 18 patients were catheterised for both painful and painless urinary retention or two PVRs of > 200 ml [13]. Ninety-seven percent of MRI positive patients had had one or more episodes of urinary incontinence but were CESI at the time of diagnosis.

CES has been divided into CESI and CESR. The distinction between CESI and CESR is that CESR is a late stage of CES with often poor outcomes, whereas CESI is associated with better outcomes particularly if treated when symptoms are modest or treated rapidly [12]. CESI has been defined as “a patient with urinary difficulties of neurogenic origin, including altered urinary sensation, loss of desire to void, poor urinary stream and the need to strain in order to micturate” [14]. This implies that there is still executive control of the bladder in the CESI patient. CESR has been defined as “painless urinary retention and overflow incontinence where the bladder is no longer under executive control” [14].

Bladder sensation is subjective, and the perception of bladder sensation is influenced by many intrinsic and extrinsic factors [15]. Examination of perineal sensation and anal tone is operator dependent. Katzouraki et al. [7] emphasised the quantitative nature of the measurement of PVR, which in general is true, but bladder scanning is operator dependent, it must be performed immediately post-void, and it can be inaccurate in patients with abdominal scarring, pregnancy, or uterine prolapse [16]. If the PVR can be measured, the patient must be able to void, and therefore these patients are not CESR [13]. In the study of Katzouraki et al. [7], most patients did not have CESR despite most reporting one or more episodes of urinary incontinence. PVR ≥ 200 ml is a sign of incomplete bladder emptying not CESR and is consistent with retention of executive control of the bladder. An ideal PVR is 0 ml, but in young adults, < 50 ml is normal; a PVR of up to 100 ml can be normal in older adults; > 200 ml is incomplete bladder emptying [15]. Anecdotally, we were aware of patients with PVRs of ≤ 200 ml who had a large compressive disc prolapse. We were also aware that the paper [7] has been used to suggest that a PVR ≤ 200 ml implies there is no risk of CES even if there are symptoms and/or signs of CES. This issue has become important in medico-legal litigation. In the case of *Jarman vs Brighton and Sussex NHS Trust* [17], it was found that there was no requirement for MR imaging in a woman with symptoms but no signs of CES and a PVR of 48 ml despite the fact that it was agreed (on the basis of subsequent progressive symptoms and signs, a positive MRI and urgent surgery) that she did have symptomatic CES at the time there was a PVR of 48 ml.

Our study was not prospective, and all our cases were self-selected on the basis of suspected malpractice; nevertheless, it was a relatively well-documented retrospective study of recent medicolegal cases. Out of the 26 patients who had post-void scans, i.e. they could void and were therefore CESI, we found 13 cases where the PVR was less than 200 ml, and the diagnosis was confirmed on MRI. Our patients are not exactly the same as the patients reported by Katzouraki et al.^7^. Case 12 had bladder symptoms but normal perineal sensation and anal tone with a positive MRI. Case 11 had bladder symptoms and subjective perineal numbness that was not confirmed on examination and normal anal tone but a positive MRI. This case would also not have satisfied the criteria for an MRI scan. Both patients (that is 2/13 or 15%) would not have satisfied the criteria for MRI of Katzouraki et al. [7]. This is also the position of the medicolegal case of Jarman [17]. These cases represent a small subset of CES cases who have symptomatic CES without signs, normal or near-normal bladder emptying with a positive MRI who do require surgery. They are similar to a previously described subset of CES patients, CES early (CESE) [18]. Early surgery is appropriate in these cases, and if the low PVR had delayed an MRI scan and had led to delayed surgery, the outcome of these patients could well have been worse than with early surgery. In our study, a further five patients had reduced perineal sensation with normal anal tone and a positive MRI where the PVR was ≤ 200 ml, which emphasises that the PVR is not a substitute for clinical assessment and a PVR ≤ 200 ml does not rule out CES where there are positive signs. Worryingly, in our medicolegal practice, we are increasingly seeing doctors say that, because the symptoms and signs of CES are not diagnostic and because the PVR is a quantitative measurement, a PVR ≤ 200 ml indicates that the patient does not have CES even in the presence of objective signs. If it was thought that a PVR ≤ 200 ml did not require an MRI (which is not what Katzouraki et al.^7^ recommended), all 13 of our cases would have been missed. We believe that in some centres, PVR is being used in an uncritical way. PVR must be considered in conjunction with the clinical symptoms and signs, not in isolation. The PVR is a potentially useful tool and further study will be important because it could be that low PVRs correlate with better bladder outcomes.

The addition of PVR to clinical assessment in the management of potential CES patients is welcome, but it must be used in conjunction with, not as a substitute for, an accurate clinical history and examination. There must be a stage in many CES cases where bladder symptoms precede incomplete bladder emptying, and this is the ideal time to make a diagnosis because outcomes are likely to be excellent. In a young patient with symptomatic lumbar degenerative disc disease and no pre-existing bladder problems, symptoms of bladder dysfunction and/or perineal sensory loss raise the question of CES even where there are no objective signs. We support the conclusions of Katzouraki et al. [7], but we believe the recommendations should be expanded to take into account patients who have symptoms but no signs of CES (Table 2). CESI patients who have objective signs of CES (including reduced perianal sensation and/or reduced VAC and/or reduced anal squeeze and/or tone should have emergency MR imaging and if the MRI is positive, emergency surgery, whatever the PVR. Any CESI patient with a PVR > 200 ml should have MR imaging regardless of objective signs given the high prevalence of CES as established by Katzouraki et al.^7^. If the MRI is positive, and there are no objective signs, these patients can probably have surgery performed urgently and that would include first thing on the following day’s emergency list. If there are symptoms but no signs and a PVR ≤ 200 ml, emergency MR imaging is not required, but we believe there should be an early nonemergency MRI to detect the small proportion of patients who have symptomatic CES only, who would benefit from surgery before they deteriorate. We would recommend MR imaging within 24 hours in these patients. This policy will not reduce the number of MRIs that are required, but it will move some into the normal working day, which is an advantage. Large numbers of these MRIs will be negative but that is the price that has to be paid for preventing potentially severe long-term harm in a small number of patients. The urgency of MR imaging in the CESR patient is more complex because it is generally accepted that more urgent surgery after CESR does not lead to better outcomes. Nevertheless, it is the case that neurological deterioration in CES is typically continuous and progressive [19, 20], and objective signs of CES continue to progress after CESR [21]. It is probably prudent to perform urgent MR imaging and then surgery on the next morning’s emergency list in CESR patients.

**Table 2:** Urgency of MRI in CES with PVR measurement

Symptoms/signs PVR	Timing of MRI
Any objective sign of CES regardless of PVR	Emergency MRI
PVR > 200 ml regardless of signs	Emergency MRI
Symptoms but no signs of CES and PVR ≤ 200 ml	MRI within 24 h

## Data Availability

All data used in this study is kept in GDPR compliant secure files and can be retrieved for review.
